# An Economic Evaluation of the Preoperative Investigations for Elective Surgical Patients at a Caribbean Tertiary Care Teaching Hospital

**DOI:** 10.7759/cureus.33528

**Published:** 2023-01-09

**Authors:** Sasha Hinds, Seetharaman Hariharan

**Affiliations:** 1 Anaesthesia and Intensive Care, Port of Spain General Hospital, Port of Spain, TTO; 2 Anaesthesia and Intensive Care, The University of the West Indies, St Augustine, TTO

**Keywords:** caribbean, costs, unnecessary tests, elective surgery, preoperative investigations

## Abstract

Introduction

Preoperative assessment using widespread laboratory investigations and ancillary tests as preoperative screening may be unnecessary and lead to an economic burden. This study aimed to determine the routine preoperative investigations performed in a tertiary care teaching hospital in the Caribbean that could be categorized as unnecessary and the costs incurred for these tests.

Methods

Patient and surgery-specific data were collected prospectively from adult elective surgery patients over a three-month period. Surgical intensity, American Society of Anesthesiologists (ASA) grade and the National Institute for Health and Care Excellence (NICE) (UK, 2016) Clinical Guideline for Preoperative Investigations were used to determine which tests to deem unnecessary. The overall economic burden of unnecessary testing was assessed.

Results

Data were prospectively collected from 636 patients during the study period. Sixty-four percent of the preoperative investigations performed were deemed unnecessary. The money spent on these unnecessary investigations amounted to $44,622. When extrapolated, this can amount to approximately $178,488 per annum. This represented 59% of the total money spent on the overall preoperative investigations performed. Relatively healthier patients (ASA I and II) had a significantly higher number of unnecessary investigations performed.

Conclusion

This study found that the majority of preoperative investigations performed routinely may be deemed unnecessary. This results in a huge economic burden on the healthcare system. There is a need to update and strictly implement clinical guidelines for preoperative investigations.

## Introduction

The preoperative assessment incorporates a detailed clinical history, physical examination, and laboratory and radiological investigations that precede anesthesia for surgical procedures. In most countries, preoperative clinical evaluation is the responsibility of the anesthetist. A vast majority of patients admitted for elective surgery undergo routine preoperative laboratory investigations and ancillary testing; however, the need for such testing is controversial [1]. Over the years, it has been well established that selective ordering of tests according to the patient’s needs is safer and more efficient for both the patient as well as the health-care system. Most investigative reports are usually ignored, and anesthesia and surgery proceed regardless of the results. Studies have shown that fewer than 5% of patients with abnormal test findings necessitate a change in clinical management before or during the procedure [2].

Preoperative assessment has the following objectives: (a) to reduce associated risks by identifying existing medical conditions; (b) to improve safety by assessing and qualifying risk; (c) to allow planning of perioperative care; and (d) to provide the opportunity for explanation to allay patient anxiety and obtain informed consent. In essence, the assessment allows planning for appropriate perioperative care, while decreasing associated adverse outcomes and facilitating the patient’s timely return to the desired level of functioning. The use of laboratory tests as an adjunct during the preoperative assessment of patients has been facilitated by medical technology that allows for rapid screening of multiple chemical factors in the blood and for automated counting of blood cells [3]. The purpose of widespread laboratory testing is also justified in the modern era of defensive practice. These are done to evaluate a known clinical condition, to identify patients at high risk of perioperative complications, to establish a baseline reference for later comparison, and to screen patients for a new disease that may affect perioperative morbidity [1,2].

However, there has been enough evidence to show that the economic burden and time consumed by widespread ancillary testing have outweighed the value added by the results obtained. Unnecessary preoperative tests can be defined as the series of tests ordered for asymptomatic individuals, in the absence of any specific clinical indication, to identify conditions undetected by clinical history and examination [2]. Such testing may not only be expensive but also lead to harm. The critique is that these tests have little impact on perioperative outcomes [4]. Studies have shown that around 60% of routine preoperative testing can be eliminated without adversely affecting patient care [1]. At a time when the cost of care and the convenience of patients are major concerns, the role of such tests as screening modalities is rightfully diminishing. Patient history, physical examination, and the judgment of the physician are replacing routine checklists as the basis for testing [5]. Laboratory investigations and ancillary tests, like all areas of medical intervention, should be undertaken on a "value-added" basis. The pre-anesthesia evaluation ought not to be utilized as a comprehensive assessment of all medical issues [5].

With this background, the current study aims to determine the cost of preoperative laboratory testing in a tertiary care teaching hospital in Trinidad and Tobago. By extension, the study will attempt to assess the economic burden of "unnecessary" testing on patients who present for elective surgical intervention at this institution.

## Materials and methods

The study was designed as a prospective observational cross-sectional study of adult patients having elective surgical procedures at the operating theatres of a tertiary care hospital in Trinidad and Tobago. Data were collected on a daily basis for three months. At the time of the preoperative assessment, one would ordinarily attain all the information needed to complete this survey, as such, no additional clinical intervention or patient exposure was required. There were no inherent risks to patients or staff members associated with the conduct of this research. The Ethics Committee of the Regional Health Authority granted approval to conduct this research with permission to waive the need for individual patient consent.

Data collected included basic demographic information (age and gender), along with parameters specific to the patient’s medical history, including a history of cigarette smoking and/or alcohol consumption, patient co-morbidities, an estimated or actual body mass index, and American Society of Anesthesiologists (ASA) grades. Other clinical data recorded included the patient’s diagnosis, the operation performed, the type of anesthetic performed, and specific laboratory or radiologic investigations conducted as part of the preoperative assessment. No specific patient identifiers were documented, and data were stored under password protection, accessible only to the primary researcher, and research supervisor.

Information was analyzed based on the most recent Clinical Guidelines published in April of 2016, by the National Institute for Health and Care Excellence (NICE), United Kingdom, outlining the preoperative tests that should be performed in adult elective surgery patients.

Patients were categorized according to the surgical intensity and then the ASA grade. Based on the NICE Clinical Guideline, the laboratory results were then categorized as necessary, to be considered, or not necessary. An additional category was included for tests that were omitted. This omitted category refers to the tests that were not conducted but should have been performed, as indicated by the NICE Clinical Guideline. The National Costing of Health Services published by the Health Economics Unit for the Ministry of Health was used to calculate the cost of preoperative tests that were performed on the patients. The cost of all investigations performed was calculated in order to highlight the cost of the preoperative investigations that were deemed "unnecessary."

Five categories of tests were identified by the analysis of the preoperative investigations using the NICE Clinical Guidelines: (i) performed, necessary tests done, also deemed as mandatory based on the NICE Guidelines; (ii) performed, to be considered - tests done are described as to be considered by the NICE Guidelines based on the patient’s co-morbidities, recent symptoms, or evaluation of risk for specific complications; (iii) performed but unnecessary - tests that were done as part of the routine preoperative evaluation but deemed inappropriate based on the NICE guidelines; (iv) not performed, appropriately omitted - investigations that were not done, as consistent with the NICE Guidelines recommendations; (v) not performed, inappropriately omitted - investigations that were omitted from the hospital preoperative evaluation, that was deemed as mandatory by the NICE Guidelines.

To evaluate the preoperative investigations that were actually performed during the three-month period, the tests were divided into categories of "appropriate" and "inappropriate."

Inclusion criteria were patients undergoing elective surgical procedures that involved anesthesia intervention in the operating theaters in general surgery, urology, gynecology (including laparoscopic procedures), obstetrics, plastic surgery, orthopedics, otolaryngology, maxillo-facial surgery, and ophthalmology.

Patients under the age of 18, patients presenting for cardiac, thoracic, neurological, and major vascular surgery, and any patient booked for an emergency operation or procedure under local anesthesia were not included in the study.

Data were entered into Microsoft Excel™ software (Microsoft® Corp., Redmond, WA), and descriptive as well as inferential statistical analyses were done. Patient gender, ASA grade, age, surgical grade, and BMI were used as categories. Statistical analyses were performed using the Pearson chi-square, Student's t-tests, and analysis of variance (ANOVA). The Statistical Package for Social Sciences (SPSS), version 21 software (SPSS Inc., Chicago, IL), was used for analyses. The statistical significance was fixed at the level of p < 0.05.

## Results

Six hundred and thirty-six (636) patients were enrolled during the three-month study period, and preoperative data were collected on all of them. Eighty-four (13%) patients had a minor surgical procedure performed, 325 (36%) had an intermediate-grade surgical intervention, and 227 (51%) patients underwent major or complex surgical procedures.

**Figure 1:**
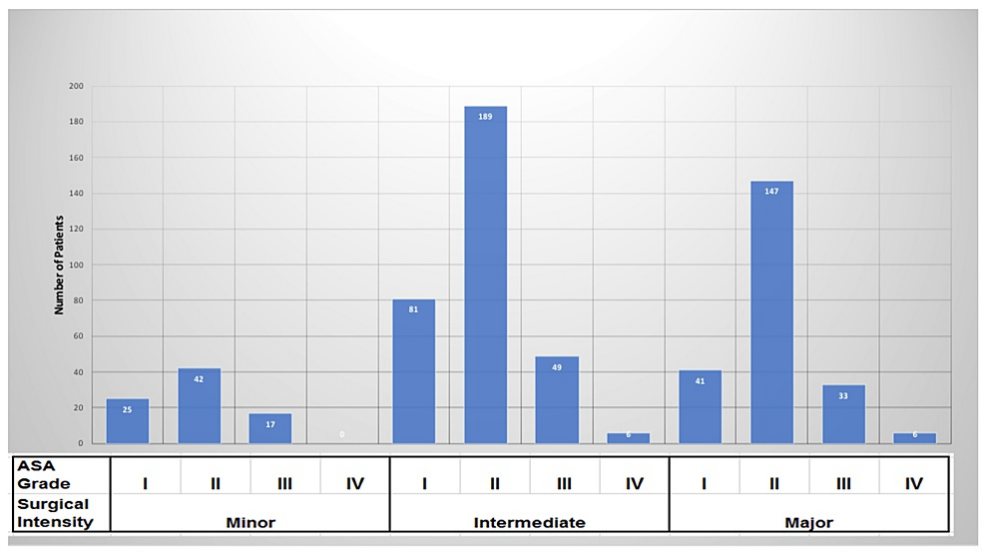
ASA grades of surgical categories of patients

Patients were categorized by ASA grade, and most cases belonged to the ASA II category. Specifically, 59% of patients belonged to ASA II and 23% to ASA I. Only 2% of patients were categorized as ASA IV. The ASA grades for each category of surgery are shown in Figure 1.

The NICE Clinical Guidelines, UK, were used to evaluate the preoperative tests performed, based on the patient's ASA grade and the surgical intensity of the proposed operation. The tests performed were identified as necessary, to be considered, or unnecessary. Further to this, tests that were not performed were also divided into two groups. These included tests that were not appropriately done and tests that should have been done but were not. The breakdown of these five categories is shown in Table 1. The tests that were unnecessary were the majority among these categories.

**Table 1:** Routine preoperative tests for elective surgery *Categories based on National Institute of Clinical Excellence (NICE), UK guidelines. CBC: complete blood count; LFT: liver function tests; PT/PTT: prothrombin time/partial thromboplastin time; RFT: renal function tests; ECG: electrocardiography; CXR: chest X-ray.

Category of tests*	CBC	LFT	PT/PTT	RFT	ECG	CXR	Total
Performed - necessary	226	0	0	240	199	14	679
Performed - may be considered	38	22	18	98	98	13	287
Performed - unnecessary	369	313	174	256	153	447	1712
Not performed - appropriately not done	2	298	441	38	142	161	1082
Not performed - necessary but omitted	1	3	3	4	44	1	56

**Figure 2:**
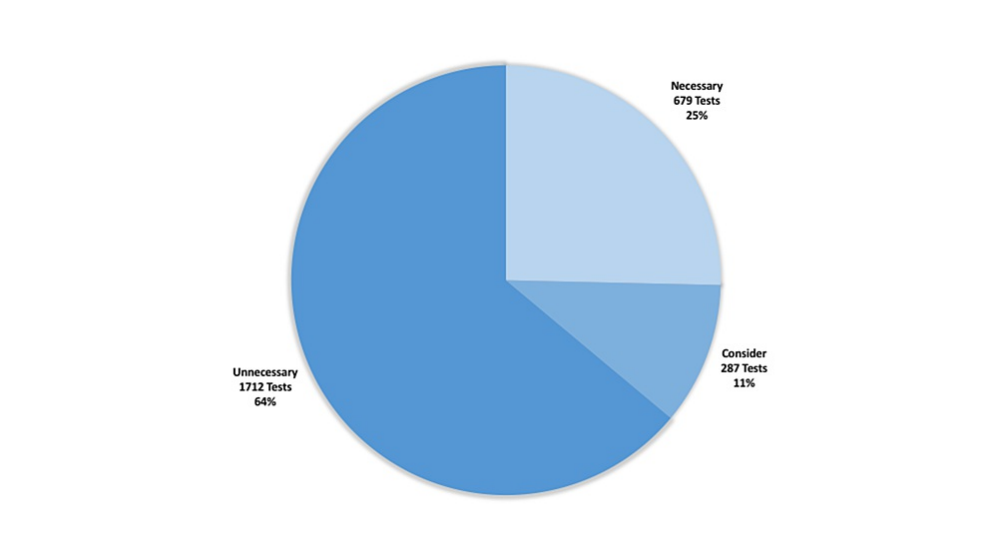
Necessity of preoperative tests according to NICE guidelines NICE: National Institute of Clinical Excellence (UK)

The investigations that were actually performed preoperatively were evaluated in three basic subcategories: tests that were appropriately done, tests that could have been considered, and tests that were inappropriately done. The distribution of these tests is shown in Figure 2. Sixty-four percent of the tests performed were found unnecessary in accordance with the guidelines.

**Figure 3:**
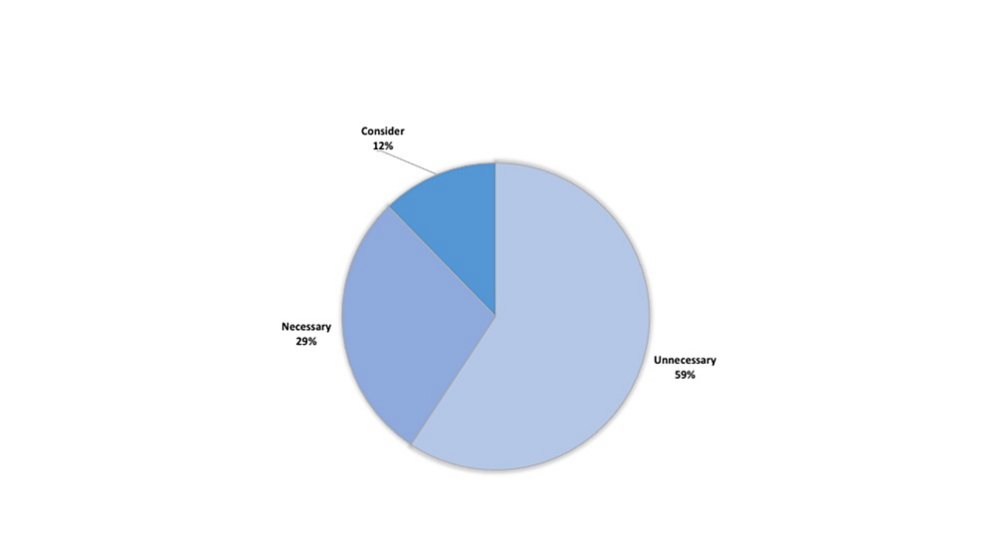
Proportion of costs of preoperative tests categorized by National Institute of Clinical Excellence (NICE), UK guidelines

A detailed analysis of the cost for each test, based on the categories delineated by the NICE Clinical Guideline, is provided in Table 2. The percentage distribution of cost is also shown in Figure 3. The greatest allocation of money was spent on unnecessary preoperative investigations that were performed, totaling $44,622. This was followed by tests that were appropriately done, which totaled $17,852.

**Table 2:** Cost for each category of preoperative tests based on NICE guidelines (USD) CBC: complete blood count; LFT: liver function tests; PT/PTT: prothrombin time/partial thromboplastin time; RFT: renal function tests; ECG: electrocardiography; CXR: chest X-ray; NICE: National Institute of Clinical Excellence, UK.

Tests	CBC	LFT	PT/PTT	RFT	ECG	CXR	Total
Performed, unnecessary	9582.98	10324.33	4518.80	8444.18	5708.95	6043.13	44622.39
Performed, necessary	5869.25	-	-	7916.41	7425.37	189.25	21400.30
Performed, vonsider	986.86	725.67	467.46	3232.53	3656.71	175.82	9245.07
Not performed, omitted	25.97	98.95	77.91	131.94	1641.79	13.58	1990.15

**Figure 4:**
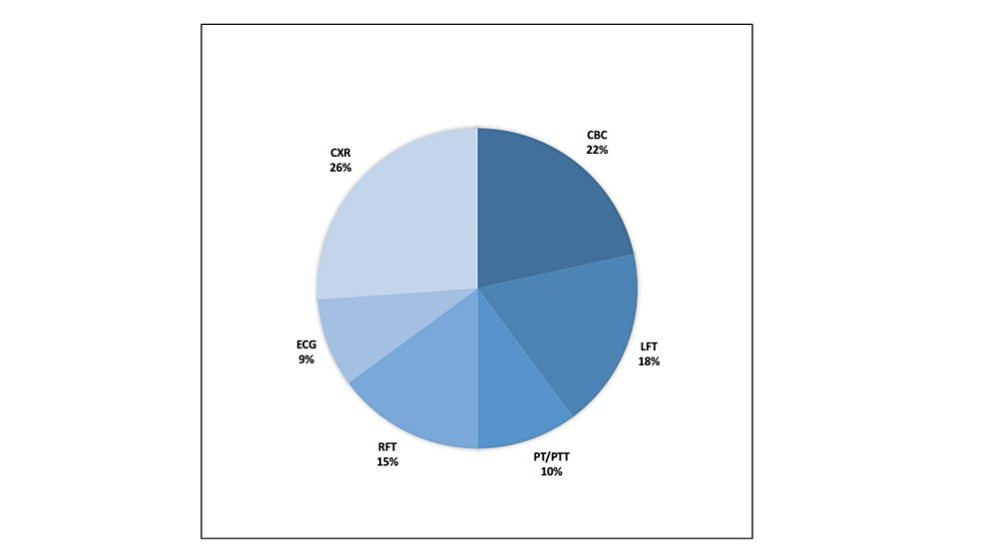
Distribution of unnecessary preoperative tests CBC: complete blood count; LFT: liver function tests; PT/PTT: prothrombin time/partial thromboplastin time; RFT: renal function tests; ECG: electrocardiography; CXR: chest X-ray.

**Figure 5:**
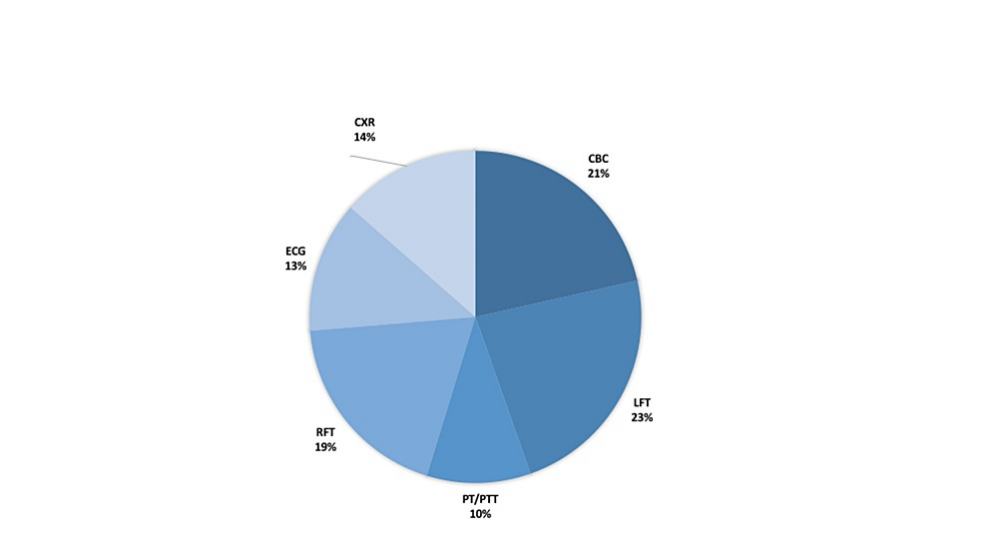
Distribution of costs of unnecessary preoperative tests CBC: complete blood count; LFT: liver function tests; PT/PTT: prothrombin time/partial thromboplastin time; RFT: renal function tests; ECG: electrocardiography; CXR: chest X-ray.

A detailed breakdown of unnecessary tests performed, the proportion of each type of test done, and the incurred costs are summarized in Figures 4-5. The largest percentage of unnecessary testing done was represented by plain chest radiographs (26%); while the largest expense was incurred by unnecessary liver function testing (LFTs), accounting for 23% of the cost ($10,324).

The comparison between the number of laboratory tests performed as well as the cost incurred did not have any statistically significant difference based on the patient’s gender and BMI. In accordance with the NICE guidelines, when the number of tests was compared between ASA grades as well as surgical intensity, there was a statistically significant difference between the categories (p < 0.001) (Table 3).

**Table 3:** Cost of unnecessary investigations by ASA grade and surgical grade

ASA	Costs (USD) mean (SEM)	Surgical grade	Costs (USD) mean (SEM)
I (n=148)	87.18 (3.60)	1 (minor) (n=84)	86.10 (4.43)
II (n=377)	71.74 (2.28)	2 (intermediate) (n=325)	84.96 (2.46)
III (n=99)	44.66 (3.05)	3 (major) (n=227)	43.06 (2.10)
IV (n=12)	21.09 (7.08)	-	-
Total (n=636)	70.16 (1.76)	-	-

Individual comparisons were made between the two categories (appropriate versus inappropriate tests) based on the patients’ ASA grades. This analysis revealed statistical significance with respect to the appropriateness of the CBC, LFT, PT/PTT, RFT, and ECG performed, and the ASA grade of the patients (p < 0.001). The same was true with respect to the CXR done (p < 0.05).

Similarly, with respect to the amount of money spent unnecessarily on CBC, LFT, RFT, ECG, and CXR, there was a statistically significant difference between the ASA grade and surgical intensity categories. Coagulation screening (PT/PTT) was the only investigation that did not reflect a significant change in the amount of money wasted based on the ASA grade.

To evaluate whether age influenced the amount of money wasted on unnecessary investigations, the patients were divided into two categories, with 65 years old as the threshold. Five hundred and twelve (512) patients belonged to the category of non-elderly (<65 years), while 124 patients fit the category of elderly (≥65 years). This analysis revealed that age was a statistically significant factor for the money wasted on RFT, PT/PTT, and CXR. The analyses showed that a significantly larger percentage of the money was expended on the category of non-elderly patients.

## Discussion

The major finding of the present study is that, despite existing hospital guidelines, there was unnecessary preoperative testing in surgical patients, especially in otherwise healthy and fit patients. Many of these patients belonged to the categories of intermediate surgical intensity and ASA grade II. When categorized in accordance with the NICE guidelines, almost two-thirds of these investigations (64%) were deemed unnecessary. A previous study conducted in the United States by Onouha et al. found that more than 70% of routine preoperative testing was unindicated, similar to the present study [6]. Another study in Brazil by Issa et al. showed that 55.8% of the ancillary investigations performed in their pre-anesthesia evaluation were deemed unnecessary [7]. These findings support the need to streamline the pre-anesthetic investigations based on an evidence-based approach that would preserve the quality of care while being cost-effective.

The high proportion of unnecessary investigations in the present study highlights the fact that voluminous information is being retrieved as part of the routine preoperative assessment for elective cases. The study hospital does not have a preoperative anesthetic assessment clinic, and as such, the laboratory investigations performed for most patients are routinely requested by junior members of the surgical team. Subsequent evaluation by the anesthesiologist usually occurs on the morning of the scheduled operation. As suggested by Korpman, some surgeons do offer a justification for routine preoperative testing with a gamut of investigations, but only because "anesthesia" wants to have them done. [8] In essence, members of the surgical team want to ensure that the case is not canceled by the anesthesiologist on the morning of the surgical procedure. In addition, within the same anesthesia department, one may encounter an occasional discrepancy in what different anesthesiologists may deem necessary preoperative investigations for a given clinical scenario. Undoubtedly, this would lead to greater confusion among the surgeons as they sought to determine which preoperative investigations had to be performed.

The total expense incurred by the tests that were deemed unnecessary amounted to $44,622 in the present study. Chandra et al. conducted a retrospective evaluation of a relatively healthy population presenting for elective general surgery and concluded that none of the preoperative investigations performed led to any additional peri-operative intervention. The cost incurred for the requested investigations was quoted at $10.81 per patient [9]. In comparison, the money spent on unnecessary investigations identified by the present study amounts to $70.16 per patient. This is well above the figure quoted by Chandra et al. and may be due to the difference in practices as well as healthcare costs across the different geographic locations.

Although the number of unnecessary investigations accounted for 64% of the total number of tests performed, the money spent on these tests represented only 59% of the total money spent on preoperative investigations within the time period evaluated. This is similar to the finding reported from Brazil, which showed that though 55.8% of the tests done were deemed "not indicated," these tests accounted for 50.8% of the total cost of investigations performed [7]. The economic analysis done by Ferrando et al. from Italy estimated that the application of preoperative guidelines would lead to a 63% reduction in the cost of preoperative tests [10]. The issue with the study hospital is that the guidelines were not stringently applied for many reasons, as mentioned earlier.

The current clinical guidelines to guide preoperative evaluation available in the study hospital were formulated and published in 2002. Strides have been made throughout the world in these two decades as to the evidence available and subsequent clinical publications that advise on appropriate preoperative investigations [11]. One significant change noted in both the US and the UK is that they have eliminated age as an independent factor used to dictate which investigations should be performed. The ASA publication, last amended in 2008, states that the more useful parameters in predicting patient outcomes are independent of the patient’s age. This is also a clear distinction noted in the 2016 update to the 2003 NICE Clinical Guideline for preoperative evaluation in elective surgery patients. The fact that the guidelines existing in the hospital are still based on age does highlight the need for a review and updating of this guideline. However, verbatim adoption of international guidelines has its own pitfalls. For example, international guidelines that have been established under different conditions may not be consistent with the local environment. For instance, Afro-Caribbean descent puts persons at higher risk of sickle cell disease, and it may be argued that doing a complete blood count as part of the routine preoperative assessment is appropriate. Also, routine visits to a wellness clinic or general practitioner are not part of the regular practice within Trinidad and Tobago. It is more likely that a patient can present for elective surgical intervention without knowledge of long-standing chronic medical conditions. These conditions can influence which investigations should be included as necessary in routine preoperative testing.

Furthermore, the international guidelines and protocols for preoperative investigation describe tests that are performed for patients who have severe cardiovascular or pulmonary pathology. Many such investigations are not readily available at the study hospital and so cannot form part of the formal assessment for most patients. These investigations include, but are not limited to, exercise stress testing, cardiopulmonary exercise testing, coronary angiography, and lung function testing.

The distribution of the money spent on each type of unnecessary investigation showed a fairly even spread. Specifically, the amount of money spent per investigation ranged from 13% to 23% of the total amount expended. Thirteen percent was spent on the electrocardiograms performed, whereas 23% could be attributed to liver function testing. The study by Chandra et al. did imply a much broader distribution of spread among the investigations deemed unnecessary. In their study, 50% of patients had a serum creatinine evaluation, whereas 100% of the patients received multiple biochemistry and hematological investigations.

Evaluation of the full spread of investigations based on the patients’ ASA grade and surgical intensity showed statistically significant differences between categories. On the other hand, there were no statistically significant differences with respect to the investigations when the patients were categorized according to demographics. These results are consistent with the current clinical guidelines that are used in the US and UK to advise which preoperative investigations ought to be performed. Most recent clinical guidelines for preoperative investigations are based on the patient’s ASA grade and proposed surgical intervention. Gender and age are no longer seen as independent factors that determine which preoperative investigations ought to be performed.

When preoperative tests were analyzed based on ASA grade, as expected, the lower the ASA grade, the higher the number of inappropriate investigations performed. Conversely, the higher the ASA grade, the lower was the number of inappropriate investigations. As a corollary, the money spent on unnecessary investigations such as CBC, LFT, RFT, ECG, and CXR in lower ASA grades was significantly higher compared to higher ASA grades.

In this era of economic scarcity, this research serves to highlight the fact that more than half the money spent on preoperative investigations for elective surgery patients may be saved. To achieve this, there is a need to effectively upgrade the 2002 Clinical Guidelines implemented in the study hospital. As early as 2000, the American Association of Family Physicians published a list of "recommended" preoperative tests before elective surgery [12]. In 2020, the total expenditure on preoperative testing in the United States alone was estimated to be 18 billion dollars [13]. The National Surgical Quality Improvement Program (NSQIP) recommendations when it was applied to low-risk ambulatory surgery patients, it was found that the ASA 1 and 2 categories may not even require preoperative laboratory testing, and the authors recommended to "re-think the routine" in such cohorts of patients [14]. The National Institute of Health and Care Excellence (NICE), United Kingdom guidelines clearly advise that the primary factors used to determine which preoperative investigations are appropriate would be the patient’s clinical history and examination, the ASA grade, and the intended surgical intervention [15]. Given the current financial constraints globally, even in developing countries such as India, the Society of Anesthesiologists has recently published very clear guidelines regarding what investigations are indicated preoperatively for different categories of surgeries and patients [16]. However, in addition to publishing guidelines, any update of the clinical guidelines must also be followed by an educational initiative. This is a crucial step that would inform staff members of both the anesthesia and surgical departments of which investigations are appropriate. Alongside the evident economic benefit, the implementation of an updated clinical guideline would also serve as a time-saving measure, which would be beneficial to all patients, physicians, and laboratory staff involved.

One limitation of the study may be the use of the Ministry of Health publication to quantify the cost of preoperative investigations performed. The costs of healthcare have always been approximate, and this has been acknowledged in all cost estimation studies. The inter-observer difference in the initial ASA grade assessment can also be a source of error. The most recent update to the ASA Classification included examples of patients in each category. Two specific examples that are not always adhered to during patient assessment would be categorizing pregnant patients as ASA II and the differentiation of specific ASA grades based on body habitus. In addition to this, at the time of data collection, there was no note of duplicate investigations that were present in the patient’s record. It is not uncommon to see more than one of the same type of investigation performed on the same patient. One typical scenario is when a patient who was postponed has returned for their intended surgical procedure but has a repeat of the preoperative laboratory tests performed within a short period of time. This may lead to an error in the calculation of the actual money spent on preoperative evaluation for such patients.

## Conclusions

Nevertheless, in summary, this study did show that the use of pre-anesthesia evaluation as a comprehensive assessment using laboratory screening is ineffective in influencing peri-operative outcomes. The laboratory investigations and ancillary tests should be undertaken only on a value-added basis since two-thirds of these tests can be potentially eliminated. In conclusion, this study clearly found that greater attention needs to be paid to the investigations being performed for elective surgery patients preoperatively. This would greatly reduce the economic burden and improve the efficiency of the preoperative assessment being performed.
